# Mobile-Based Analysis of Malaria-Infected Thin Blood Smears: Automated Species and Life Cycle Stage Determination

**DOI:** 10.3390/s17102167

**Published:** 2017-09-21

**Authors:** Luís Rosado, José M. Correia da Costa, Dirk Elias, Jaime S. Cardoso

**Affiliations:** 1 Fraunhofer Portugal AICOS, Rua Alfredo Allen 455/461, 4200-135 Porto, Portugal; dirk.elias@fraunhofer.pt; 2 Instituto Nacional de Saúde Dr. Ricardo Jorge, Rua Alexandre Herculano 321, 4000-055 Porto, Portugal; jose.costa@insa.min-saude.pt; 3 INESC TEC (Institute for Systems and Computer Engineering, Technology and Science) and Department of Electrical and Computer Engineering of the Faculty of Engineering, University of Porto, Rua Dr. Roberto Frias, 4200-465 Porto, Portugal; jaime.cardoso@inesctec.pt

**Keywords:** image analysis, malaria, computer-aided diagnosis, microscopy, mobile devices

## Abstract

Microscopy examination has been the pillar of malaria diagnosis, being the recommended procedure when its quality can be maintained. However, the need for trained personnel and adequate equipment limits its availability and accessibility in malaria-endemic areas. Rapid, accurate, accessible diagnostic tools are increasingly required, as malaria control programs extend parasite-based diagnosis and the prevalence decreases. This paper presents an image processing and analysis methodology using supervised classification to assess the presence of malaria parasites and determine the species and life cycle stage in Giemsa-stained thin blood smears. The main differentiation factor is the usage of microscopic images exclusively acquired with low cost and accessible tools such as smartphones, a dataset of 566 images manually annotated by an experienced parasilogist being used. Eight different species-stage combinations were considered in this work, with an automatic detection performance ranging from 73.9% to 96.2% in terms of sensitivity and from 92.6% to 99.3% in terms of specificity. These promising results attest to the potential of using this approach as a valid alternative to conventional microscopy examination, with comparable detection performances and acceptable computational times.

## 1. Introduction

Between 2010 and 2015, malaria mortality rates fell by an estimated 29% globally and by 31% in the African region due to the scale-up of malaria interventions. Despite this remarkable progress, the global tally of malaria in 2015 was still 212 million new cases and 429,000 deaths, it being estimated that malaria surveillance systems detected only 19% of the cases that occurred globally. Most of these deaths occurred in the African region (92%), but it is estimated that nearly half of the world’s population is at risk of malaria, being considered endemic in 91 countries [1]. Across Africa, millions of people still lack access to the tools they need to prevent and treat the disease. Prompt diagnosis can not only prevent the development of severe malaria, but also reduce the length of time that patients carry malaria parasites (MPs) in their blood, which in turn reduces the risk of onward transmission [1].

A recent report [2] considers that current funding distribution on malaria control commodities (US $1.6 billion in 2014) is not addressing the fundamental weaknesses in health systems of developing countries, suggesting that innovative ways may be required to rapidly expand access to malaria interventions. It is worth underlining that the mobile phone is currently Africa’s most important digital technology [3], and just as African telecommunications largely skipped over landline infrastructure and went straight to mobile phones, some experts say African medicine can skip over centralized labs [4]. Moreover, the combination of mobile devices with image processing for malaria diagnosis can bring several advantages, like potentially reducing the dependence of manual microscopic examination, which is an exhaustive and time-consuming activity and requires considerable expertise of the laboratory technician.

This paper presents an image processing methodology using supervised classification to analyze microscopic images of malaria-infected thin blood smears, by assessing the presence of MPs and determining the species and life cycle stage. One of the main contributions of this work is the usage of images acquired exclusively with smartphones. This differentiation factor is directly related with the integration of this methodology in a mobile-based framework currently being developed, which aims to support the pre-diagnosis of malaria in rural areas. Moreover, the proposed approach was designed taking deeply into account the clinical knowledge of each particular structure we aim to detect (e.g., morphology, staining behavior and expected inner structures).

## 2. Malaria Disease Characterization

This section gives an overview of essential concepts for understanding malaria, namely the characterization of MPs in terms of growth stages and species, as well as the recommended malaria diagnostic methods. In order to keep the text self-contained, we are going to revisit the characterization of the malaria disease as presented in [5].

MPs are blood parasites that are transmitted to humans through the bite of an infected mosquito, passing over three different growth stages: (i) Trophozoites: the most commonly-seen stage, which can vary from small to quite large within red blood cells (RBCs). Trophozoites have usually only one chromatin dot, but two are commonly seen in *P. falciparum* species. The cytoplasm morphology takes different shapes, ranging from demarcated fine rings to irregular and amoeboid forms; (ii) Schizonts: Trophozoites mature into schizonts through multiple divisions of the parasite into daughter cells called merozoites. Each merozoite is in fact a new parasite, with its own chromatin body and cytoplasm. The infected RBCs eventually bursts, allowing the new merozoites to travel within the bloodstream to infect new RBCs; (iii) Gametocytes: some trophozoites differentiate into sexual erythrocytic stages called gametocytes, usually about the size or bigger than RBCs. Gametocytes present variable morphologies, from round to banana-shaped, depending on the species. In terms of biological classification, MPs are protozoan parasites of the genus *Plasmodium*. Five species of *Plasmodium* can infect and be transmitted by humans: *P. falciparum*, *P. vivax*, *P. ovale*, *P. malariae* and *P. knowlesi*.

**Figure 1:**
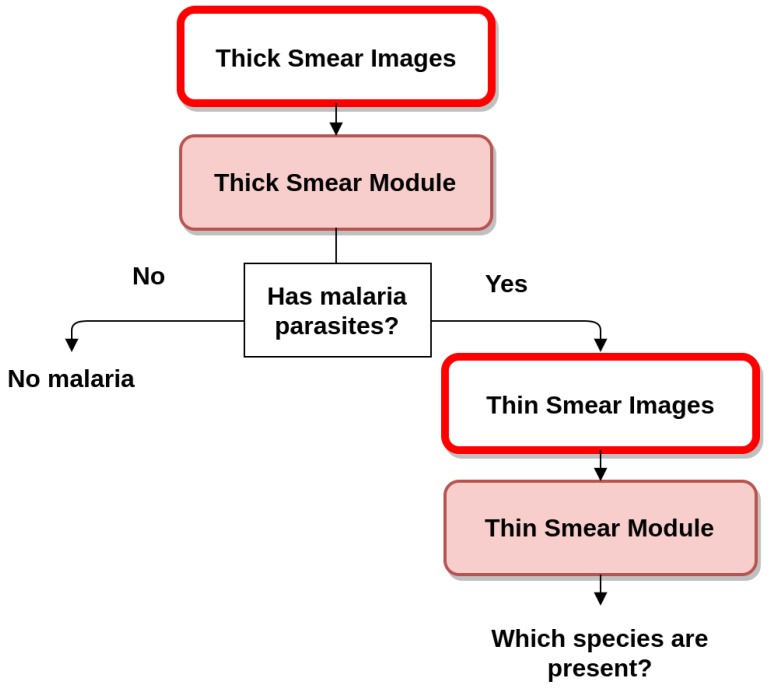
Blood smear analysis flow for both quantification and species/life cycle stage identification.

Since 2010, WHO has recommended that all persons with suspected malaria should undergo malaria diagnostic testing, by either microscopy or rapid diagnostic tests (RDTs). Microscopy examination consists of preparing a blood smear, staining it (most often with Giemsa stain) and examining it through a microscope. This process remains the mainstay of malaria diagnosis in most large health clinics and hospitals. However, microscopy-based diagnosis in rural areas is frequently non-existent or inadequate in terms of quality due to scarce resources (equipment and trained staff). When stained MPs are spotted by the microscopist, the diagnosis of malaria is confirmed by identifying the respective stage, species and infection density [6]. Microscopic examination can be made through the usage of thin and thick blood smears. While the thin smear consists of a single layer of RBCs, the thick smear is six- to 20-times thicker, allowing for a greater volume of blood to be examined. Thus, thick smears are firstly used to check the presence of MPs, while thin smears are subsequently analyzed for the identification of MP species. The conventional flow for both quantification and species/life cycle stage identification is presented in Figure 1.

Malaria diagnosis through RDTs is accomplished by detecting specific malaria antigens in a person’s blood. However, the use of the RDT does not eliminate the need for malaria microscopy for two main factors: (i) the RDT may not be able to detect some infections with low parasite density; (ii) the currently approved RDT in the U.S. only detects two different malaria antigens (one is specific for *P. falciparum*, and the other is found in all four human species of malaria). Thus, microscopy is further needed to determine the parasite species and to quantify the proportion of RBCs that are infected, which is an important prognostic indicator [7].

## 3. Related Work

Several image processing approaches have been recently proposed for the identification of MPs in thin blood smears. One of the first approaches was proposed by Zou et al., 2010 [8], through the usage of an improved circle Hough transform to detect RBCs. By analyzing the RGB (red, green, blue) color space, the authors observed that nucleated components present distinctively high intensity values in the B channel and simultaneously low intensities in the G channel. Therefore, an approach to stretch the contrast of nucleated objects was proposed based on the observed differences between these two color channels.

In order to obtain a more detailed analysis of infected RBCs, Anggraini et al., 2011 [9], proposed a methodology focused on detecting three regions inside these structures: MP nucleus, MP cytoplasm and RBC cytoplasm. For each region, image features based on area ratios and range of intensities were extracted. The detection of infected RBCs using a Bayes decision rule classifier achieved a sensitivity (SE) of 92.59% and a specificity (SP) of 99.65%, but only 60 and 20 images were used for training and testing, respectively.

A different approach for RBCs segmentation was proposed by Malihi et al., 2013 [10], which merges Otsu’s segmentation with edge detection via Canny’s method. After passing a hole-filling process, image features were extracted for each segmented structure, more specifically in terms of gradient, texture, histogram and area granulometry. Five different classifiers were tested, the best results being achieved by a kNN (k-nearest neighbors) with 80% of SE and 95.5% of SP.

Later on, a k-means clustering for MPs segmentation was proposed by Khan et al., 2014 [11]. This method has the particularity of being based on the usage of the b*-color channel of the CIE (Commission International de l’Élairage) L*a*b* (lightness, green/red coordinate, blue/yellow coordinate) color space. A total of 118 Leishman-stained microscopic images were used, results being reported of 76% and 60% for SE and SP, respectively. However, the proposed approach simply consists of classifying the images as infected/not infected, thus not giving information about the number of correctly-identified MPs in each image, nor the respective species and growth stage.

In another work by Nugroho et al., 2015 [12], segmentation of MPs was made through a k-means algorithm. Using histogram-based texture features, the authors aimed to detect the parasites in three different life cycle stages using a multilayer perceptron with backpropagation algorithm, with reported SE of 81.7% and SP of 90.8%. However, only *P. falciparum* was considered, with results obtained using a limited dataset of 60 manually-cropped sub-images.

Nanoti et al., 2016 [13], used k-means clustering applied on RGB, HSI and L*a*b color spaces for MPs’ segmentation, followed by various textural and shape features. A total of 300 images with 386 annotated MPs were used for the classification in 13 classes (12 classes for three life cycle stage for each of the four malaria species and one class for non-infected blood samples). However, the classes distributions were not detailed, i.e., the number of parasites for each species-stage combination. Two classifiers were used, kNN and SVM (support vector machines), with the best performance for kNN with accuracy (AC) of 90.2% and SE of 90.2%. Unfortunately, only the overall performance is given, not being detailed for each species-stage combination.

More recently, Yang et al., 2017 [14], developed a low-cost image-based cytometer for scoring and classifying MPs’ stages, with the innovative aspect of comparing the obtained parasitemia results with flow cytometry. The authors used an adaptive thresholding approach for segmentation and an SVM with a linear kernel for classification, with reported high specificity, sensitivity and negligible false positives (~0.0025%). However, several limitations can be identified for the proposed approach: Only 25 positive images were used to train the classifier; the classification is based on a simple three-dimensional feature space, namely the average pixel intensity; several parameters are defined in terms of the number of pixels, thus requiring manual user calibration for images with different pixel resolutions; Stage differentiation is exclusively based on the area occupied within the RBCs, the detection of gametocytes not being considered; and finally, this study only considered *P. falciparum*.

Despite these promising results during the past few years, most of the proposed methodologies are based on two main requirements unsuitable for most malaria-endemic areas: (i) images acquired under well-controlled conditions; and (ii) the need for proper microscopic equipment. Both criteria are difficult to accomplish in those areas where this type of equipment and the know-how to maneuver it are scarce or nonexistent. As an alternative, here we present a different methodology for automated analysis of malaria-infected thin blood smears by using images exclusively acquired with low-cost and accessible tools such as smartphones. Despite not being the main focus of this article, it should be noted that this work represents only a component of a mobile-based framework for MPs’ detection currently being developed, which will be briefly described in Section 4.

In terms of the selected approaches for image processing and machine learning, we go beyond the state-of-the-art and achieve a more robust and consolidated methodology that tackles the issue of identifying different MPs’ species-stage combinations on the same image. For that purpose, some of the successful outcomes previously reported were tested, adapted and integrated in our methodology, along with new proposed processing steps, which will be detailed in Section 5. Finally, the proposed approaches are greatly inspired by the in-depth clinical knowledge of each particular species-stage combination we aim to the detect (e.g., morphology, staining behavior and expected inner structures).

## 4. Mobile-Based Framework for Malaria Parasites Detection: An Overview

**Figure 2:**
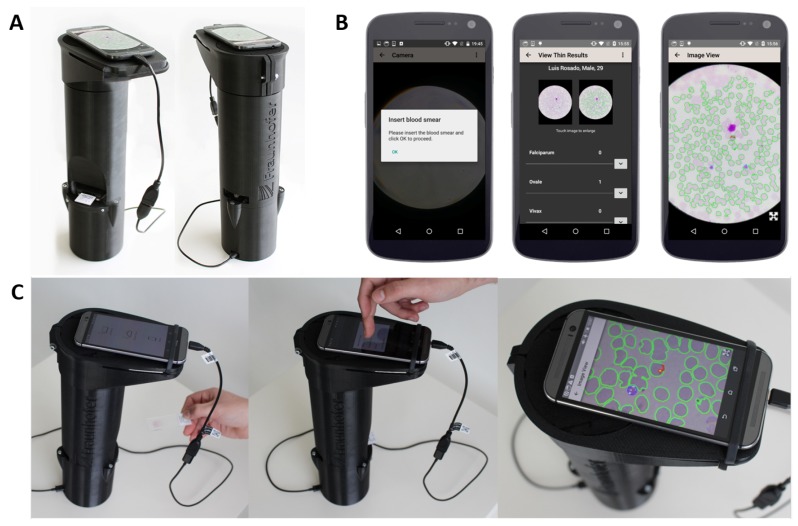
Mobile-based framework for malaria parasites’s detection: (**A**) μSmartScope with smartphone attached and blood smear inserted; (**B**) smartphone application screenshots; (**C**) exemplificative usage of the solution (from left to right): (i) blood smear insertion; (ii) start image acquisition through the smartphone app; and (iii) visual feedback of the automated detection.

The work reported in this paper represents only a component of a mobile-based framework for MPs’ detection currently being developed, which is composed by three main components (see Figure 2):

I μSmartScope: an inexpensive alternative to the current microscopes that can easily be adapted to a smartphone and used in the field. This gadget guarantees the required 1000× magnification, and the smartphone camera is used to capture images. Moreover, it uses a self-powered motorized automated stage system, in order to move the blood smear and allow the automatic capture of several snapshots of the sample [15];
II Image processing and analysis: consisting of the automated detection of MPs via computer vision and machine learning approaches, on microscopic blood smear images acquired using I. This component consists of two main modules, as detailed in Section 2:
  (a) Thick smear module to detect the presence of MPs on thick blood smears [16].
  (b) Thin smear module for the determination of MPs species and life cycle stage (main focus of this article).
III Smartphone application: envisioned to be used by technical personnel without specialized knowledge in malaria diagnosis. The user collects and prepares a blood sample of the patient, introducing it in a slot of I. Using the companion mobile application, installed in the smartphone that is coupled to I, the user can take pictures of the blood smear using the smartphone’s camera, being subsequently analyzed by II, so the correct procedures and medication can be administered.

## 5. Methodology

**Figure 3:**
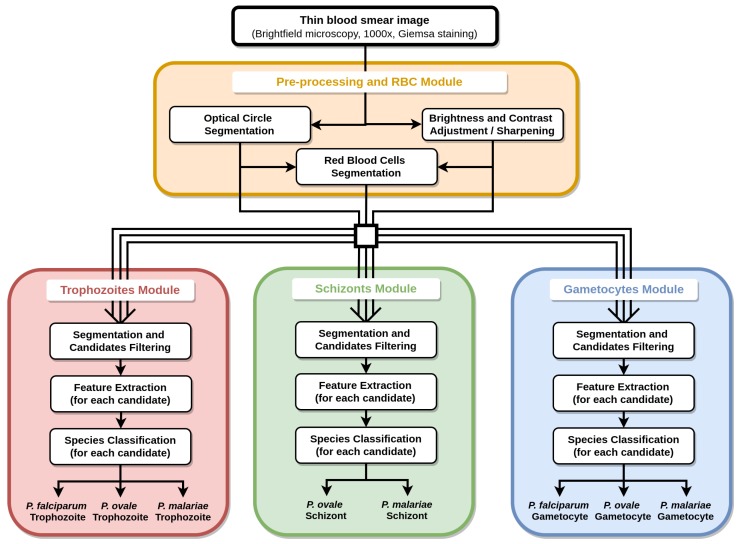
Diagram of the proposed methodology for the automatic analysis of thin smear images.

The proposed methodology for the automatic determination of MPs species and life cycle stage on mobile-acquired thin smear images can be divided into 4 main blocks (see Figure 3): (i) pre-processing and RBC module; (ii) trophozoites module; (iii) schizonts module; and (iv) gametocytes module.

### 5.1. mThinMPs Database

**Figure 4:**
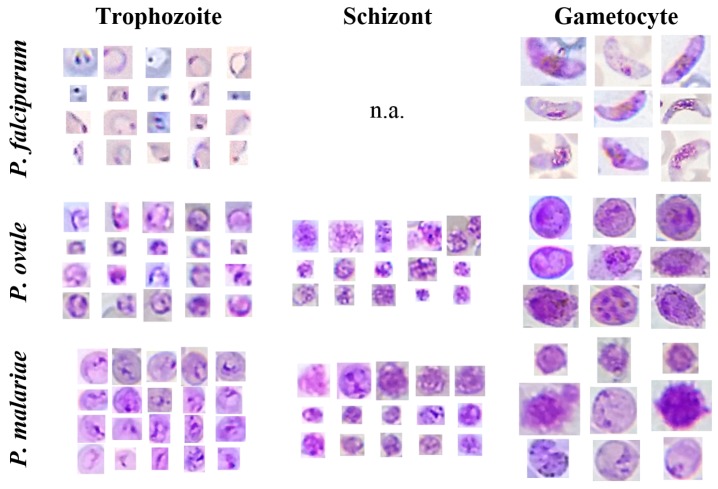
Illustrative examples of different MPs species and life cycle stages from the Mobile Thin Smear Malaria Parasites (mThinMPs) database.

The Mobile Thin Smear Malaria Parasites (mThinMPs) Image Database is an unpublished dataset acquired from 7 different thin blood smears infected with different MPs (see Figure 4). The blood smears were supplied by the Instituto Nacional de Saúde Dr. Ricardo Jorge, Portugal, and the images acquired using the μSmartScope prototype coupled to a smartphone (see Section 4). Two different smartphones were used, an HTC One S and an LG Nexus 5, with image resolutions ranging from 1944 × 2592 to 1840 × 3264 pixels. This image database contains a total of 566 microscopic images. The images were manually annotated (bounding boxes) by an experienced parasitologist from the Infectious Diseases Department of Instituto Nacional de Saúde Dr. Ricardo Jorge, with a total of 1127 identified MPs. A detailed description of the manual annotations by species and life cycle stage is depicted in Table 1.

It is worth noting that no representatives of *P. falciparum* schizonts are present in the dataset since they are rarely seen in peripheral blood. Moreover, this database does not include examples of *P. vivax*, since all specimens were obtained via African clinical partners, and *P. vivax* has practically no clinical incidence in Sub-Saharan Africa.

### 5.2. Pre-Processing

The pre-processing procedure includes two main steps: (i) brightness and contrast adjustment and (ii) sharpening.

#### 5.2.1. Brightness and Contrast Adjustment

**Figure 5:**
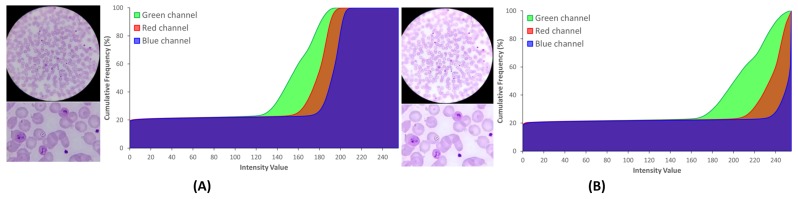
Effect of brightness and contrast adjustment, with cumulative histograms: (**A**) original image; (**B**) processed image after α and β correction, followed by mean-shift filtering.

The images acquired with the μSmartScope will have a circular region of interest (ROI) at the center of the image, a region from now on termed the optical circle (see Figure 5). Outside this region, the image is expected to be black, while inside the optical circle, the background is expected to be near white.

**Figure 6:**
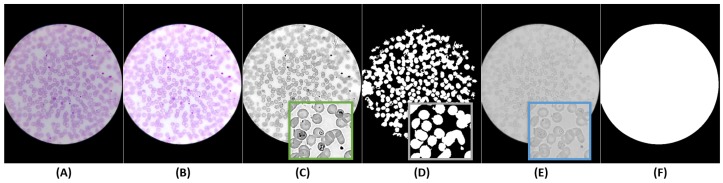
Pre-processing: (**A**) original image; (**B**) brightness and contrast adjustment; (**C**) sharpening applied over green channel of adjusted image; (**D**) RBCs; segmentation applied over the sharpened image; (**E**) blue channel of the original image; (**F**) optical circle segmentation applied over the blue channel of the original image.

The brightness and contrast were adjusted through a commonly-used procedure that applies a constant gain α and bias β to the original image. From the histogram perspective, α and β will operate as the color range amplifier and range shift, respectively. These parameters are computed automatically by assuming that the desired histogram range is 255, and only intensities with more than 1% frequency are considered to define the minimum and maximum intensity values used to stretch the histogram (see Figure 5). As a consequence of this process, in Figure 5B a higher range of intensities where the G channel is clearly separated is observable. This channel was considered in previous works [8,17] the most distinctive RGB channel for stained components, a fact that our research also confirms (see Figure 6C). Therefore, an increased demarcation of the G channel as a result of the contrast adjustment is a clearly positive aspect, since it will later facilitate the segmentation process of the stained components.

On the other hand, the cumulative histogram also shows that the B channel becomes much more polarized around two major clusters near 0 and 255. Previous works also confirm [8,17] that stained components on the B channel present a significantly lower intensity demarcation, making this channel highly interesting for the optical circle segmentation. Looking at B channel image in Figure 6E, it becomes clear that these two major clusters represent in fact the regions inside and outside that region. To finalize, a smoothing procedure using a mean shift filtering was applied [18]. We chose this particular filter due to its edge-preserving characteristics, which was shown to greatly facilitate the following segmentation process by simultaneously preserving the edges of stained components and homogenizing the stain color intensities.

#### 5.2.2. Sharpening

The goal of this pre-processing step is to increase the sharpness of the stained components by using an unsharp masking procedure. Particularly, the unsharped mask was obtained by blurring the target image using a Gaussian filter with a fixed window radius of 15 and combining it with the original image according to the weights of Equation (1):

(1) $$\;I_{Shar}=1.5\times I_{Green}-0.5\times I_{Gau}-\left(0.75\times I_{Green}⊙\;0.2\times I_{Lap}\right),$$

where the image $I_{Green}$ is the green channel of the brightness and contrast adjustment output and $I_{Shar}$ the sharpened image. It is worth noting that a Laplacian component was also added to the sharpening procedure. The unsharp mask can cause artifacts on edge borders, so the Laplacian component is responsible for avoiding double edges. This component was obtained through an element-wise multiplication (⊙) of the original image with the Laplacian of the original image using the following kernel:$\left[\begin{array}{ccc} 0 & 1 & 0\\ 1 & -4 & 1\\ 0 & 1 & 0 \end{array}\right]$.

### 5.3. Segmentation and Filtering

The segmentation and filtering component of the proposed methodology can be divided into 5 main blocks: (i) optical circle segmentation; (ii) RBCs’ segmentation; (iii) trophozoites’ segmentation; (iv) schizonts’ segmentation; and (v) gametocytes’ segmentation.

The most relevant difference between the currently most popular segmentation approaches and the adaptive thresholding approach proposed in this work is the usage of the in-depth clinical knowledge of each particular structure. Particularly, we took advantage of its known morphology, staining behavior and expected inner structures to customize the respective segmentation procedures. With that purpose, we started by mapping the expected maximum length of those structures in μm (see Table 2), in order to adapt the respective segmentation methods accordingly.

The optical diameter $D_{circle}$ depicted in Table 2 represents the maximum diameter of the optical circle mask, for which the real size was calculated in a previous work [15]. The microscopic images have a fixed magnification of 1000×, so $D_{circle}$ is used as a metric reference that makes this adaptive segmentation approach independent of the image resolution. Particularly, $D_{circle}$ ratios were later used as the initial reference for tuning the window sizes of the different segmentation and area filtering algorithms.

#### 5.3.1. Optical Circle Segmentation

According to the contrast analysis of Section 5.2.1, only the B channel of the original image was used for the optical circle segmentation (see Figure 6E,F). Since this is a fairly trivial segmentation task, we opted for a simple approach that allows a fast computational performance. A median filter with a large window factor was firstly applied to eliminate the inner structures, and the segmentation was performed using Otsu’s method, a well-known histogram shape-based image thresholding routine. The remaining inner structures inside the optical circle were then removed using a flood fill algorithm.

#### 5.3.2. RBCs Segmentation

As previously explained, the proposed segmentation methods for the different structures were independently customized according to the known clinical knowledge. However, all of the segmentation methods have the particularity of being based on the same adaptive thresholding approach. Considering the pre-processed image $I_{Shar}$, the corresponding segmented image $I_{Seg}$ is obtained according to Equation (2):

(2) $$I_{Seg}\left(x,y\right)=\left\{\begin{array}{cc} 0 & \mathrm{if}I_{Shar}\left(x,y\right)>T_{Shar}\left(x,y\right)\\ 255 & \mathrm{otherwise} \end{array}\right.,$$

where $T_{Sharp}$ is the mean intensity value of the square region centered on the pixel location (*x*, *y*) with a side value of $W_{Side}$ minus the constant *C*. For the RBCs’ segmentation, we used C=3, and $W_{Side}$ is given by Equation (3), with a scale factor $S_{Factor}=1.5^{-1}$.

(3) $$W_{Side}=D_{circle}\times S_{Factor}$$

The optical circle mask is then subtracted to eliminate segmented regions outside of the optical circle, and a closing morphological operation with an elliptical structuring element of size 3 is then applied. Finally, the RBC candidates pass through an area filtering process with $A_{RBC}^{Min}=D_{circle}\times 2^{-1}$ and $A_{RBC}^{Max}=D_{circle}\times 100$ used as the minimum and maximum area thresholds, respectively. As a side note, the defined value for $A_{RBC}^{Max}$ is much higher than initially expected. This is related with the significant overlap of RBCs commonly verified, which results in agglomerated structures that should not be discarded, since the RBCs’ mask will be further used to filter certain MPs candidates that develop exclusively inside RBCs.

#### 5.3.3. Trophozoites’ Segmentation

**Figure 7:**
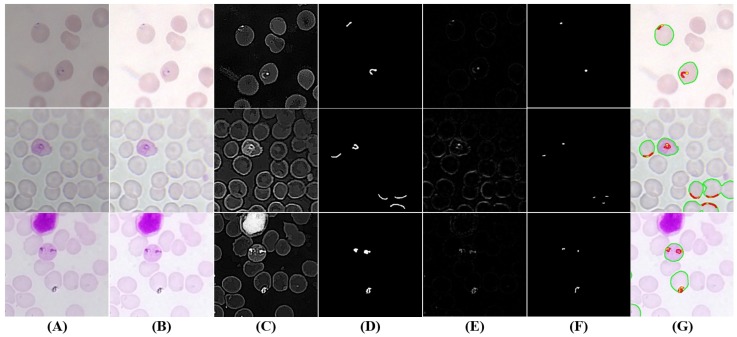
Examples of trophozoites ring stage candidates: (**A**) original image (cropped ROI); (**B**) brightness and contrast enhancement; (**C**) cytoplasm grayscale sharpening; (**D**) cytoplasm segmentation and filtering; (**E**) chromatin grayscale sharpening; (**F**) chromatin segmentation and filtering; (**G**) final candidates (cytoplasm in red; chromatin in yellow; RBC with candidate inside in green).

**Figure 8:**
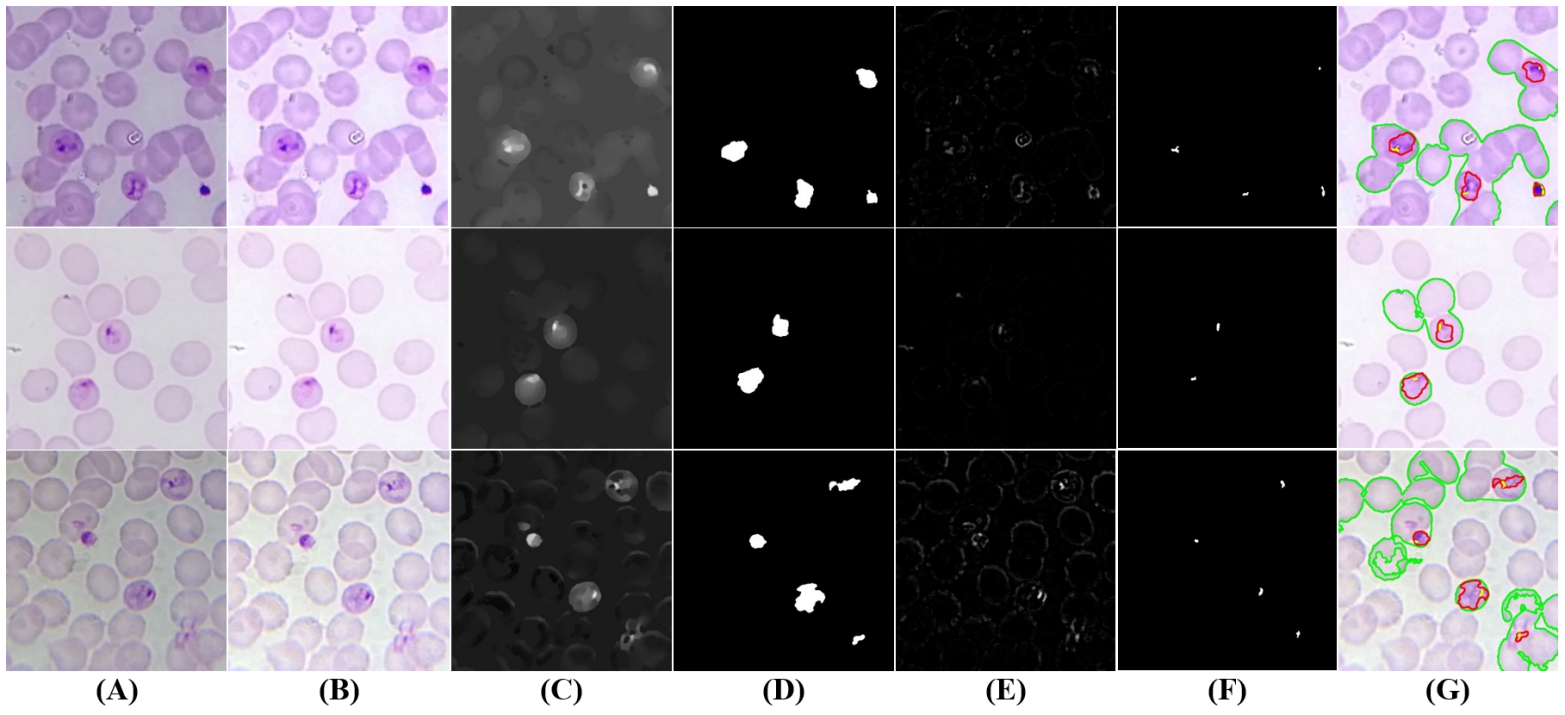
Examples of mature trophozoite stage candidates: (**A**) original image (cropped ROI); (**B**) brightness and contrast enhancement; (**C**) cytoplasm grayscale sharpening; (**D**) cytoplasm segmentation and filtering; (**E**) chromatin grayscale sharpening; (**F**) chromatin segmentation and filtering; (**G**) final candidates (cytoplasm in red; chromatin in yellow; RBC with candidate inside in green).

When merozoites infect an RBC, they develop to form ring-stage trophozoites, which then progress to mature trophozoites. While the chromatin dots usually are relatively similar along trophozoites’ growth, there are significant differences in terms of cytoplasm morphology, as depicted on Figure 7 and Figure 8. Thus, we propose a single approach to segment trophozoites chromatin dots, but the cytoplasm segmentation follows different paths for ring and mature stages.

Chromatin dots segmentation: The chromatin dot is a part of the parasite nucleus, usually round in shape, and stains red with Giemsa. It resembles to a sharp small dot, so the algorithm consists of creating a local differences mask. The $I_{Shar}$ is blurred using a median blur filter with a $W_{Side}$ obtained via Equation (3) and consistent with Table 2 ($S_{Factor}$ = $7^{-3}$). The small structures will disappear with the blurring process, among them the chromatin dots. The original image is then subtracted to the blurred image, and a 3-channel local differences mask is obtained (see Figure 7E and Figure 8E). To binarize this mask, the maximum value in each pixel position along the 3 channel is selected, and the adaptive thresholding explained previously is applied with the same $S_{Factor}$ used in the blur process and C=−14. To obtain the final chromatin dot mask, an area filtering process is then applied using $A_{Chrom}^{Min}=D_{circle}\times 9^{-3}$ and $A_{Chrom}^{Max}=D_{circle}\times 8^{-2}$ as the minimum and maximum area thresholds, respectively (see Figure 7F and Figure 8F).

**Figure 9:**
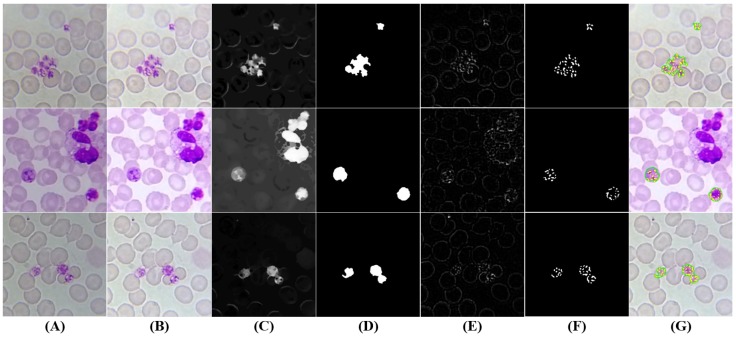
Examples of schizonts candidates: (**A**) original image (cropped ROI); (**B**) brightness and contrast enhancement; (**C**) cytoplasm grayscale sharpening; (**D**) cytoplasm segmentation and filtering; (**E**) merozoites’ chromatin grayscale sharpening; (**F**) Merozoites’ chromatin segmentation and filtering; (**G**) final schizonts candidates (cytoplasm in green; chromatin in yellow).

**Figure 10:**
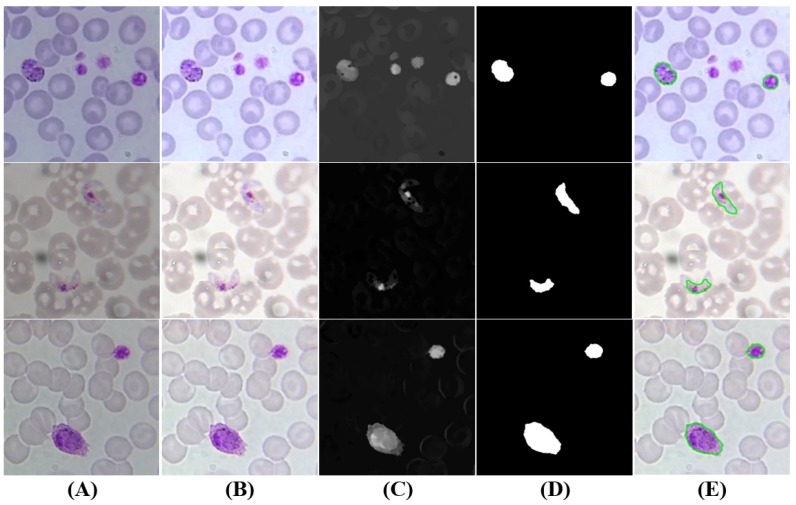
Gametocytes candidates: (**A**) original image (cropped ROI); (**B**) brightness and contrast enhancement; (**C**) grayscale sharpening; (**D**) segmentation and filtering; (**E**) final candidates (at green).

Ring-stage cytoplasm segmentation: A preliminary cytoplasm segmentation mask is firstly obtained by tuning the threshold parameters of the adaptive thresholding approach previously explained. Since the cytoplasm stain contrast and expected dimensions are, respectively, significantly lower and higher than chromatin, the thresholding parameters were adapted accordingly (C=25, $S_{Factor}=2^{-2}$). It should be noted that this preliminary segmentation mask successfully includes most ring- and mature-stage cytoplasms, but they are usually merged with parts of RBCs. In order to refine the preliminary segmentation mask and remove these undesired RBC regions, the first consideration to be taken into account is that trophozoites have a darker stain intensity than RBCs, which is the reason why they are visible inside the RBCs. The second consideration is that stain intensity highly depends of the smear preparation procedure, as visible in Figure 7, Figure 8, Figure 9 and Figure 10. Therefore, in order to obtain an enhanced version of $I_{Shar}$ (termed $I_{Shar}^{Tropho}$) that is as independent as possible from the smear stain intensity and simultaneously highlights trophozoites, we start by using the previously-obtained RBCs mask and compute the mean intensity value inside RBCs structures. The regions of $I_{Shar}$ outside the preliminary segmentation mask are then set to the computed mean value, a process that brings the background pixels intensities closer to the RBCs intensities. This image is then used to obtain the $I_{Shar}^{Tropho}$ by subtracting the B channel from the G channel, a process that highlights the trophozoites’ cytoplasm according to the contrast analysis made in Section 5.2.1 (see Figure 7C). The final ring cytoplasm binary mask (see Figure 7D) is then obtained via adaptive thresholding of $I_{Shar}^{Tropho}$ (C=−30; $S_{Factor}=1^{-1}$), followed by an area filtering process ($A_{Ring}^{Min}=D_{circle}\times 2^{-2}$; $A_{Ring}^{Max}=D_{circle}\times 5^{-1}$).

Mature-stage cytoplasm segmentation: The cytoplasm segmentation for the mature stage is quite similar to the one described for the ring stage, only changing the used adaptive threshold parameters and area thresholds, as a consequence of the described morphological differences. Particularly, the preliminary cytoplasm mask for the mature stage is obtained with C=3 and $S_{Factor}=1^{-1}$, while the final mature cytoplasm binary mask with C=−40 and the same $S_{Factor}$. Finally, an area-filtering process is applied ($A_{Mature}^{Min}=D_{circle}\times 1^{-1}$; $A_{Mature}^{Max}=D_{circle}$), followed by a hole-filling procedure (see Figure 8C,D).

Masks merging and candidates filtering: After obtaining the chromatin and cytoplasm segmentation masks separately, a mask merging process is responsible for merging these masks consistently and according to the expected morphological characteristics. Considering that trophozoites always appear inside RBCs on thin smears, the previously-generated RBCs’ segmentation mask was firstly used to filter both trophozoites’ segmentation masks, being only considered for further processing the candidate structures inside segmented RBCs. Moreover, all trophozoites must have at least one chromatin dot inside, so the cytoplasm candidates with no chromatin candidates inside are discarded, for both ring- and mature-stage masks. On the other hand, chromatin candidates that are not inside either a ring or mature cytoplasm candidate are also discarded. Finally, the filtered cytoplasm masks for ring and mature stages were summed into a single cytoplasm mask.

#### 5.3.4. Schizonts’ Segmentation

Trophozoites develop into schizonts, which divide several times to produce new merozoites. The infected RBCs eventually burst, allowing the new merozoites to travel within the bloodstream to infect new RBCs. Thus, the proposed approach divides the schizonts segmentation task into two main steps: merozoites chromatin and schizonts cytoplasm segmentation.

Merozoites’ chromatin segmentation: While the merozoites’ cytoplasm is quite hard to differentiate in the acquired images, the schizonts’ chromatin is very distinctive and similar to trophozoites’ chromatin in terms of morphology and stain intensity. Consequently, we reused the approach described in Section 5.3.3 for chromatin dots’ segmentation, with two small adjustments: (i) decreasing the *C* adaptive threshold parameter (C=−5), since merozoites’ chromatin stain intensity can be less demarcated when compared to trophozoites’ chromatin; and (ii) increasing the maximum area threshold ($A_{Chrom}^{Max}=D_{circle}\times 1.3^{-1}$), since the maximum dimension of merozoites’ chromatin can be bigger (see Figure 9E,F).

Schizonts’ cytoplasm segmentation: The maturation from trophozoite to schizont is a gradual process, so it is natural that trophozoites and schizonts share similarities in terms of visual appearance. However, since merozoites grow and duplicate inside schizonts’, a bigger cytoplasm is expected for schizonts when compared with mature trophozoites. Thus, the approach described on Section 5.3.3 for mature trophozoites’ cytoplasm segmentation was reused for schizonts’ cytoplasm segmentation (see Figure 9C,D), the maximum area threshold only being tuned ($A_{Schizont}^{Max}=D_{circle}\times 3$).

Mask merging and candidate filtering: In order to merge the information of these two different segmentation masks, we started by using the merozoites’ chromatin mask to filter the schizonts’ cytoplasm candidates, i.e., only the cytoplasm candidates with at least one merozoite chromatin dot inside are considered for further processing. On the other hand, merozoites’ chromatin candidates that are not inside a cytoplasm candidate are also discarded (see Figure 9G).

#### 5.3.5. Gametocytes Segmentation

Despite being originated via distinct cellular differentiation mechanisms, there are a few visual resemblances between mature schizonts and gametocytes in terms of dimensions and stain intensity. Nevertheless, two denoted differences between schizonts and gametocytes were used to adapt the approach described in Section 5.3.4 to segment gametocyte candidates (see Figure 10): (i) the average stain intensity of gametocytes is much more demarcated than schizonts, so the respective *C* adaptive threshold parameter in the final segmentation stage was tuned accordingly (C=−20); (ii) gametocytes are on average bigger than schizonts, so the minimum and maximum area thresholds were increased ($A_{Gametocyte}^{Min}=D_{circle}\times 4^{-1}$; $A_{Gametocyte}^{Max}=D_{circle}\times 4$). There is however an exception to this observation, namely the fact that *P. falciparum* banana-shaped gametocytes may be smaller than the respective schizonts, but this particular case is not relevant for our study since *P. falciparum* schizonts are rarely seen in blood samples.

### 5.4. Feature Extraction

Instead of limiting any subsequent analysis to an initial choice of a limited set of features and since previous guidelines on high quality images may not hold for the images acquired with the proposed low-cost system, we decided to record a wide range of features. The considered list of image features was based on the outcomes of an extensive review of all of the significant features already proposed on the literature for malaria parasite detection on thin smears [5], merged with features so far not used for this purpose. We use a total of 152 image features, grouped into 3 major groups: geometry, color and texture features (see Table 3). The usage of the L*C*h${}^{\circ }$ (lightness, chroma, hue) color space for the color group features is directly related to its particular characteristics of being device independent and designed to match human perception.

It should be noted that another differentiation factor of our work is the usage of the clinical knowledge for each particular species-stage combination also in terms of the extracted image features. In Section 5.3, we described the extraction of more than one segmentation mask for some life cycle stages, for instance by separately segmenting the inner structures commonly present in that stage. In fact, the morphology and staining behavior of those inner structures can be highly relevant for the correct visual characterization of a specific species-stage combination, so we want to supply this detailed information to the classification model. The next sub-sections detail the image features extracted or each particular stage.

#### 5.4.1. Trophozoites’ Features

Each trophozoite candidate will be composed by a trophozoite cytoplasm candidate and one chromatin dot candidate. Despite the event of two chromatin dots associated with a specific cytoplasm candidate being uncommon, only one of those chromatin candidates will be considered for feature extraction. The selection of the representative chromatin candidate is made according to the following criterion: since chromatin dots have a demarcated circular shape, we select the chromatin candidate with the minimum elongation feature value (the ratio between the minimum and maximum distance from the center of mass to the boundary). A total of 314 features is then extracted for each trophozoite candidate: the 152 image features referred to in Table 3, extracted independently for the correspondent trophozoites’ cytoplasm and chromatin dot candidates; 10 ratios between specific cytoplasm and chromatin features (area, maximum and minimum diameter, perimeter, convex hull area, bounding box area, relative difference of the C* and h channels’ mean values, the difference of the coefficient of variation of the C* and h channels).

#### 5.4.2. Schizonts’ Features

Each schizont candidate will be composed by a schizont cytoplasm candidate and at least one merozoite chromatin dot candidate. Unlike trophozoites, schizonts can present 10–36 chromatin dots in the erythrocytic stage, each one belonging to a different merozoite. Due to the high number of expected chromatin candidates, the selection of a single candidate (as described above for the trophozoites) appears to be unsuitable for the schizonts’ scenario. Taking this into account, a total of 204 features is extracted for each trophozoite candidate: the 152 image features for the schizont cytoplasm; averages of the 48 color features detailed in Table 3 extracted for each merozoite chromatin candidates; 4 color ratios between cytoplasm and chromatin features (the difference of the C* and h channels’ mean values, the difference of the coefficient of variation of the C* and h channels).

#### 5.4.3. Gametocytes Features

Unlike the other life cycle stages, the gametocytes’ segmentation procedure results in a single binary mask. Thus, the 152 images features referred to above are extracted for each gametocyte candidate.

### 5.5. Classification

For machine learning training purposes, each observation was labeled according to the manual annotation, i.e., labeled as MPs of that specific species-stage combination if the overlap coefficient (also known as the Szymkiewicz–Simpson coefficient) between the region of interest of the candidate and the manual annotation is higher than 0.75. For each species-stage combination, a two-class SVM classifier with an RBF kernel was used to create a classification model, with the hyperparameters γ and *C* tuned accordingly. The classification model workflow that is responsible for connecting the eight generated classification models is explained in Section 6. It should be noted that no feature selection method was applied, since the performance bounds on which the SVM maximal margin is based are independent of the feature space dimensionality. This means that a good generalization performance can be achieved with huge dimensional feature spaces, but requires a careful tuning of the hyperparameters.

#### 5.5.1. Data Augmentation

**Figure 11:**
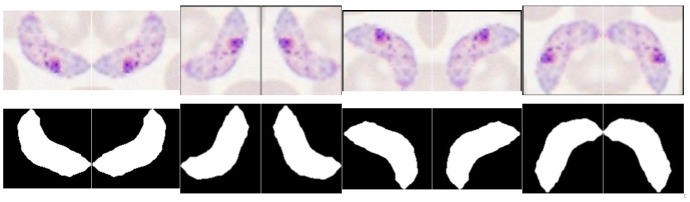
Illustrative examples of the data augmentation procedure.

Data augmentation is commonly used to prevent overfitting and improve performance in imbalanced class problems. For our dataset, we verified a significant imbalance between MP and non-MP classes for all species-stage combinations (see Table 4). Thus, 7 new observations were created for each candidate labeled as MP through sequential 90${}^{\circ }$ rotations and respective mirroring of the RGB and binary mask images (see Figure 11). The feature extraction for each replica was made according to the procedure described in Section 5.4. It should be noted that the index of the original observation for each generated replica was saved, in order to ensure during the training process that the original candidate and all the respective replicas are included in the same train/test set after the random split of the hold outs.

#### 5.5.2. SVM Hyperparameters Selection

Choosing optimal hyperparameter values is a crucial step during SVM design, usually involving the usage of specific performance metrics. The importance of the performance metric selection under this context can not be overstated, since it will greatly impact the behavior of the obtained classification model. It is worth noting that the usage of different performance metrics yields different trade-offs, so the requirements that are demanded for a specific classification scenario should always be taken into account during this process. For our particular scenario, we looked for metrics that primarily aim to improve the performance with respect to the target positive class (MP). However, due to our class-imbalanced data context, an acceptable performance to detect the non-target class (non-MP) was also required. Thus, we opted for the combination of two different metrics that weight sensitivity with specificity and precision, respectively: the informedness metric (also called Youden’s index) and the F_1_ score. In particular, a customized grid-search approach to obtain the best γ and *C* was implemented, according to the following steps:

1. All of the features were normalized between [−1,1].
2. Defining the search interval for each hyperparameter, namely $\gamma \in [2^{-15},2^{3}]$ and $C\in [2^{-5},2^{15}]$.
3. Defining the maximum number of rounds for the individual optimization of each hyperparameter ($HP_{Round}$), namely $HP_{Rounds}^{Total}=5$. In each $HP_{Round}$, the target hyperparameter $HP^{Target}$ is optimized, while the other parameter remains with a fixed value. The process starts with the γ optimization and fixed C=1.
4. Defining the maximum number of rounds for the overall optimization of both hyperparameters ($Overall_{Round}$), namely $Overall_{Rounds}^{Total}=3$. It should be noted that each $Overall_{Round}$ corresponds to the individual optimization of each $HP^{Target}$, as described in the previous step.
5. For each $HP_{Round}$, a hold out strategy is used by randomly splitting the dataset into training and test sets. A total of 20 hold outs was applied for each tested [γ;C] combination, with 90% of the observations of both classes on the training set and the remaining used for testing purposes.
6. The best hyperparameters combination $\left[\gamma^{Best};C^{Best}\right]$ is selected according to the performance metrics criteria described in Algorithm 1. This new proposed criteria aims to ensure that we select a classification model that has a balanced performance between the detection of both classes, even in data imbalance contexts. Particularly, this criteria allow the selection of a [γ;C] that implies a decrease in one of the two considered metrics (when compared with the $\left[\gamma^{Best};C^{Best}\right]$ found so far), but only if there is a performance improvement of the second metric that is twice greater than the performance loss suffered by the first.
7. At the end of each $HP_{Round}$ and while $HP_{Rounds}^{Total}$ is not achieved, the search interval is updated. Particularly, the search interval is updated to $HP\in [HP^{Best}\times 0.5,\;Hp^{Best}\times 1.5]$, being the main goal of this step the fine tuning of $HP^{Target}$.
8. A total number of 50 values of $HP^{Target}$ for each search interval was tested. Due to the large value range and possible different orders of magnitude, the logarithmic scale was used to select equally-spaced values on the search interval. Considering the search interval $HP\in [HP^{Begin},HP^{End}]$, the value on the *i*-th position is given by:
  (4) $$\;HP_{i}=10^{log_{10}\left(HP_{Begin}\right)+log_{10}{\left(\frac{HP_{End}}{HP_{Begin}}\right)}^{i/\#HP_{values}}},$$
  where $\#HP_{values}$ is the maximum number of considered values for $HP^{Target}$ under the considered search interval, with $i=\{0,1,\ldots ,\#HP_{values}\}$.

**Algorithm 1:** Performance metrics criteria.
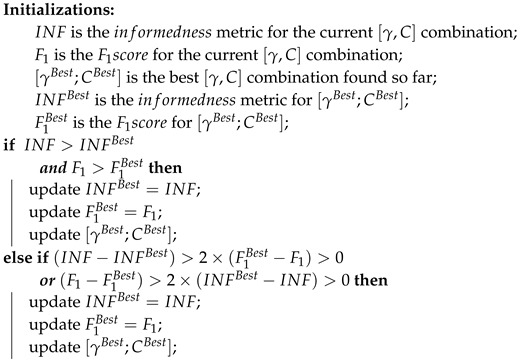


## 6. Results and Discussion

The results for the segmentation step are depicted in Table 4. Despite the significant imbalance between MP and non-MP classes for all stages, it should be noted that only 61 out of 1127 ground truth annotations do not have a segmented binary structure associated, thus not being considered for further SVM classification. In terms of the parameters required for the segmentation approach, the selection criteria were deeply influenced by the particularities of each stage in terms of morphology, staining behavior and expected inner structures. It worth noting that these parameters do not have a fixed value; they are automatically calculated for each new image through the extraction of the respective $D_{circle}$ value. Thus, the proposed segmentation approach requires little user calibration and is independent of image size.

**Figure 12:**
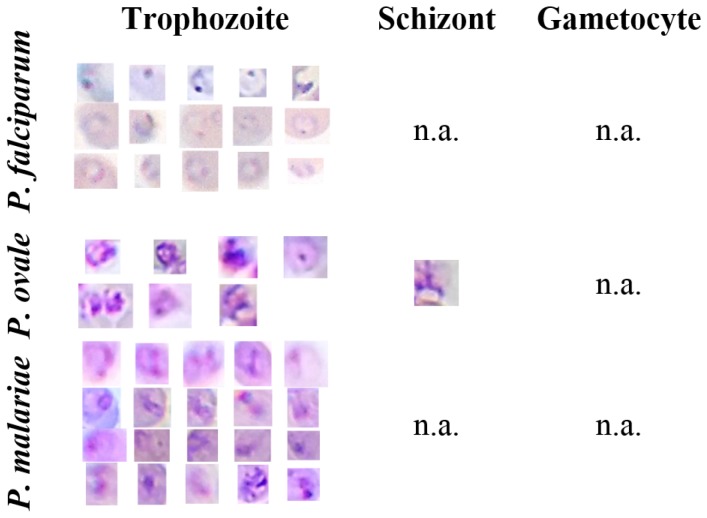
Examples of false negatives’ candidates for different species and life stages after segmentation and filtering.

Illustrative examples of false negatives can be consulted in Figure 12, where the apparent unfocus observable on several of those annotated regions can potentially explain the segmentation procedure failure. However, this does not necessarily mean that the respective entire image of those annotations was globally unfocused, since it is not possible to obtain images with maximal focus in the whole optical circle area due to the lens constraints of μSmartScope [15].

On the other hand, examples of candidates correctly segmented and labeled as MPs can be seen in Figure 4. At this stage, we opted to tune the adjustable segmentation and filtering parameters to catch most of the ground truth annotations, being aware that this option would certainly increase the number of false positives. Our strategy consisted of tackling the class-imbalanced problem later in the classification step, by using the previously described data augmentation and SVM hyperparameters’ selection procedures.

The classification results are presented in terms of five metrics: (1) sensitivity (SE), i.e., the percentage of candidates correctly classified as MPs; (2) specificity (SP), i.e., the percentage of candidates correctly classified as non-MPs; (3) accuracy (AC), i.e., the percentage of candidates correctly classified overall; (4) informedness (INF), i.e., the arithmetic mean between sensitivity and specificity; and (5) F_1_ score (F_1_), i.e., the harmonic mean between sensitivity and precision. In Table 5 are detailed the results after machine learning classification for the different species-stage combinations.

**Figure 13:**
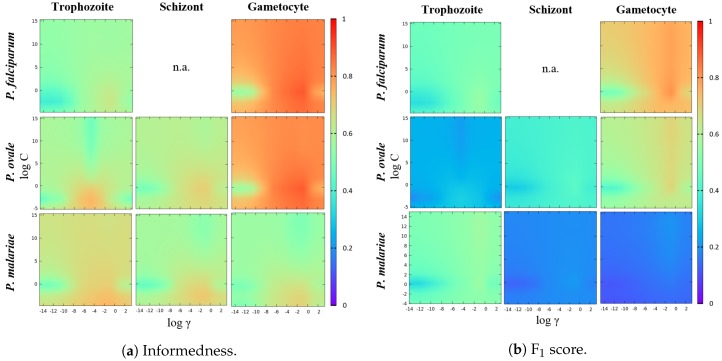
Heat maps of the SVM parameters’ selection process for each species-stage combination.

Considering our class-imbalanced data context, not only the acceptable performances obtained, but also a clear consistency and balance between SE, SP and AC values for the different species-stages combinations should be highlighted, which is a direct result of the implemented SVM classifier training involving data augmentation and a customized SVM hyperparameters’ selection procedure. The importance of using a performance metrics criterion that merges the INF and F_1_ metrics is illustrated in the heat maps of Figure 13. Firstly, it is noticeable that the [γ;C] regions that boost the INF metric are clearly more demarcated when compared with the best [γ;C] regions for the F_1_ metric. However, for highly class-imbalance scenarios (such as the ones depicted for *P. malariae* schizonts and gametocytes), there is an accentuated decrease in the F_1_ metric caused by a precision metric decrease. In other words, this is caused by the significant increase of the false positives when compared with the number of true positives. Thus, taking into account the F_1_ metric in this type of scenario might be crucial for the overall performance of the classifier, since the sacrifice of the detection of a few true positives can lead to a significant decrease of the detected false positives.

It is worth mentioning that the reported classification performance has the potential to become more robust if the known limitations of the current version of the mThinMPs database are addressed, namely the imbalanced number of manual annotations for the different species-stage combinations and the lack of *P. vivax* examples.

### Classification Models’ Workflow

**Figure 14:**
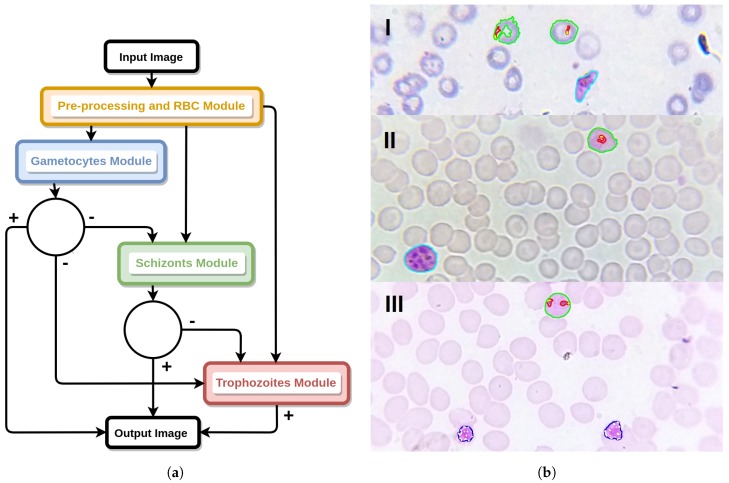
Classification models workflow. (**a**) Diagram of the classifier models workflow for the detection of multiple species-stage combinations in a single image. (**b**) Illustrative examples with detection of: (I) *P. falciparum* trophozoites and gametocyte; (II) *P. ovale* trophozoite and gametocyte; (III) *P. malariae* trophozoites and schizonts.

In order to allow the detection of multiple species-stage combinations in a single image, we had to design a single processing flow that connects the generated machine learning classification models. A diagrammatic representation of the process is illustrated in Figure 14.

In terms of overall performance by life cycle stage, the best detection results are achieved for gametocytes, being simultaneously the stage with bigger MPs structures. Thus, the designed workflow starts with the execution of the gametocytes module, with all of the candidates classified as gametocytes added to the output image. In order to avoid the classification of the same structure (or parts of it) as belonging to different species-stage combinations, the gametocytes mask is used to filter the candidates segmented in the schizonts module, i.e., if the overlap coefficient between a classified gametocyte structure and a schizont candidate is higher than 0.75, that schizont candidate is discarded. A similar procedure is applied to the trophozoites module, where the trophozoites candidates are filtered using the structures classified as schizonts and gametocytes. In the event of a particular structure being classified as positive for different species under the same life cycle stage, the distance to the separating hyperplane is used as the tie-breaking criterion, i.e., we select the species that is further apart from the decision boundary.

The proposed methodology was implemented in C++ using the Open Source Computer Vision (OpenCV) library Version 3.2.0 [20]. In terms of performance, the registered average computational time for the classification of the 566 images included in the mThinMPs image database was 16 s per image, running on an Intel^®^ Core™ i7-4790 CPU with 3.60 GHz (with OS Ubuntu 14.04 LTS). Moreover, the memory allocation of the proposed methodology was also analyzed using the Valgrind profiling tool, with maximum memory peaks detected always below 240 MB for the images used in this study. It should be noted that the obtained computational times and memory allocations can be considered suitable for scenarios where access to similar or better computational resources is available (including via cloud computing). On the other hand, considering the current reality of malaria-endemic rural areas, the execution of the developed image processing module exclusively on mobile devices is also currently being considered by our group. However, given the (still) comparably lower computational resources of these devices, code optimizations will certainly be required in order to obtain comparable processing times.

## 7. Conclusions and Future Work

In this work, an image processing methodology using supervised classification to analyze microscopic images of malaria-infected thin blood smears is presented. Particularly, the proposed approach assesses the presence of MPs and determines the species and life cycle stage. One of the main contributions of this work is the usage of images acquired exclusively with smartphones, with the consequent customization of the proposed methodology for images with such characteristics. This differentiation factor is directly related with its integration in a mobile-based framework currently being developed, which aims to support the pre-diagnosis of malaria in rural areas.

Given the lack of freely available image datasets, a new mobile thin smear malaria parasites (mThinMPs) image database was specifically created and used in this study. By comparing the results obtained with manual annotations of a microscopy specialist, we validated the performance and robustness of the proposed methodology to detect eight different species-stage combinations, with an automatic detection performance ranging 73.9–96.2% in terms of sensitivity and 92.6–99.3% in terms of specificity. These promising results attest to the potential of using this approach as a valid alternative to conventional microscopy examination, with comparable detection performances and acceptable computational times.

To achieve these results, the importance of a newly-proposed performance metrics criterion for SVM hyperparameters’ selection should be highlighted, which merges the informedness and F_1_ score metrics. Moreover, we also took into consideration the importance of providing detailed visual outputs, so another innovative aspect of this work is the identification of relevant inner structures of MPs like chromatin dots and merozoites, since we consider that useful and easily interpretable outputs are of extreme importance in the acceptance and dissemination of computer-aided diagnosis systems.

As future work, we aim to complement the mThinMPs image database with more annotations, namely: (i) add illustrative cases of *P. vivax* in all three growing stages; and (ii) increase the number of examples for the already considered species-stages combinations, with special focus on those less represented (e.g., *P. malariae* schizonts and gametocytes). The extended mThinMPs database is envisioned to support the future improvements of the proposed methodology in terms of detection performance and robustness. As a final note, this work represents only a component of a mobile-based framework for MPs’ detection currently being developed. Thus, we aim to integrate this methodology into the referenced framework, with the ultimate goal of creating a system that: (i) provides an effective pre-diagnosis of malaria in medically-undeserved areas; (ii) is low cost and mobile based; (iii) is easy to use, even for non-experts in microscopy.

## Figures and Tables

**Table 1:** MPs’ manual annotations by species and life cycle stage in the mThinMPs database.

	Trophozoites	Schizonts	Gametocytes
***P. falciparum***	585	n.a.	58
***P. ovale***	122	80	62
***P. malariae***	164	27	29

**Table 2:** Maximum length and respective $D_{circle}$ relative ratios of RBCs’ and MPs’ structures [19].

	Length (μm)	Approximate $D_{\mathrm{circle}}$ Ratio
$D_{\mathrm{circle}}$	215	-
**RBCs**	7~8	$3^{-2}$~$4^{-2}$
**Trophozoites**	1~7	$4^{-3}$~$4^{-2}$
**Schizonts**	5~10	$2^{-2}$~$5^{-2}$
**Gametocytes**	7~14	$3^{-2}$~$7^{-2}$

**Table 3:** Summary of the extracted image features.

Group	Family	Channels	Features
***Geometry***		Binary	Maximum Diameter ^a^, Minimum Diameter ^a^, Perimeter ^a^, Eccentricity, Convex Hull Area ^a^, Area ^a^, Elongation Bounding Box Area ^a^, Solidity, Extent, Circularity, Elliptical Symmetry, Principal Axis Ratio, Radial Variance, Asymmetry Indexes/ Ratios, Compactness Index, Irregularity Indexes, Bounding Box Ratio, Lengthening Index, Equivalent Diameter ^a^, Asymmetry Celebi.
***Color***		C* and h${}^{\circ }$ (from L*C*h${}^{\circ }$)	Mean ^b^, Standard Deviation ^b^, L1 Norm ^b^, L2 Norm ^b^ Entropy ^b^, Energy ^b^, Skewness ^b^, Kurtosis. ^b^
	Discrete Fourier Transform	Grayscale	Mean, Standard Deviation, Maximum, Minimum.
***Texture***	Gray Level Run Length Matrix	Grayscale	Short run emphasis ^c^, long run emphasis ^c^, run percentage ^c^, long run high grey level emphasis ^c^, low grey level runs emphasis ^c^, high grey level runs emphasis ^c^, short run low grey level emphasis ^c^, short run high grey level emphasis ^c^, grey level non-uniformity ^c^, long run low grey level emphasis ^c^.
	Gray Level Co-occurrence Matrix	R, G, B (from RGB)	Energy ^c^, Entropy ^c^, Contrast ^c^, Correlation ^c^, Maximum probability ^c^, Dissimilarity ^c^, Homogeneity ^c^.
	Laplacian	Grayscale	Mean, Standard deviation, Maximum, Minimum.

^a^ Feature divided by *D_circle_*, in order make it independent of image size; ^b^ feature computed independently for each channel, as well as for the grayscale masks that result by folding and subtracting the region of interest of each channel according the major and minor axis of inertia; ^c^ feature computed for the following directions: 0^∘^, 45^∘^, 90^∘^ and 135^∘^.

**Table 4:** Results after the segmentation step for each MP stage.

	True Positives	False Positives	False Negatives
** Trophozoites**	811	13,701	60
** Schizonts**	106	5733	1
** Gametocytes**	149	4190	0

**Table 5:** Results after machine learning classification for each species-stage combination.

	SVM Parameters	Sensitivity	Specificity	Informedness	F_1_ Score	Accuracy
***P. falciparum*** **Trophozoites**	γ = $5.46^{-2}$ *C* = $2.00^{-3}$	73.9%	97.0%	70.9%	60.0%	96.1%
***P. falciparum*** **Gametocytes**	γ = $1.01^{-1}$ *C* = 1	94.8%	99.3%	94.1%	87.4%	99.2%
***P. ovale*** **Trophozoites**	γ = $1.31^{-5}$ *C* = $1.01^{-3}$	84.6%	97.0%	81.6%	34.8%	96.9%
***P. ovale*** **Schizonts**	γ = $4.45^{-2}$ *C* = 1.55	82.7%	97.9%	80.6%	52.6%	97.7%
***P. ovale*** **Gametocytes**	γ = $1.08^{-1}$ *C* = 1	96.2%	99.0%	95.2%	77.1%	99.0%
***P. malariae*** **Trophozoites**	γ = $3.17^{-2}$ *C* = 1	82.0%	99.1%	81.1%	63.5%	98.9%
***P. malariae*** **Schizonts**	γ = $2.97^{-2}$ *C* = 1	87.8%	96.5%	84.3%	25.9%	96.5%
***P. malariae*** **Gametocytes**	γ = $6.33^{-2}$ *C* = $8.13^{-5}$	94.9%	92.6%	87.5%	18.8%	92.6%

## Abbreviations

The following abbreviations are used in this manuscript:

MPs	Malaria parasites
RBCs	Red blood cells
RDTs	Rapid diagnostic tests
SE	Sensitivity
SP	Specificity
AC	Accuracy
INF	Informedness
F_1_	F_1_ score
ROI	Region of interest
SVM	Support vector machines
kNN	K-nearest neighbors
RGB	Red, green, blue
CIE	Commission International de l’Élairage
L*a*b*	Lightness, green/red coordinate, blue/yellow coordinate
L*C*h${}^{\circ }$	Lightness, chroma, hue
HSI	Hue, saturation, intensity
